# Evaluation of the efficacy of 20% IR3535^®^ with a sustained-release formulation and 25% DEET insect repellents against mosquitoes in a field setting in Ghana

**DOI:** 10.1186/s13071-025-06946-1

**Published:** 2025-10-07

**Authors:** Mufeez Abudu, Andy Asafu-Adjaye, Joseph Harold Nyarko Osei, Kwadwo Kyereme Frempong, Osei Kwaku Akuoko, Sellase Pi-Bansa, Mavis Ofei, Helena Anokyewaa Boakye, Jane Ansah-Owusu, Sandra-Candys Adwirba Arkorful, Michelle Ayuritolya Asigbaase, Christopher Nii Laryea Tawiah-Mensah, Beatrice Greco, Delalih Manteau, Thalita Jesus, Daniel Oppong, Andy Mahler, Daniel Adjei Boakye, Samuel Kweku Dadzie

**Affiliations:** 1 Department of Parasitology, Noguchi Memorial Institute for Medical Research, College of Health Sciences, University of Ghana, Legon, Ghana; 2 Global Health Institute, MerckKGaA, Darmstadt, Germany; 3 Electronics, Darmstadt, MerckKGaA Germany; 4 LivFul Inc, Alpharetta, USA

**Keywords:** IR3535^®^, Repellent, DEET, *Anopheles gambiae* s.l., Repellency, Ghana

## Abstract

**Background:**

Personal protection with topical skin repellents has been advocated for use against vector-borne diseases. This study compared the efficacies of a 20% IR3535^®^ lotion with Staytec technology formulation and 25% DEET lotion in repelling mosquitoes in two rural communities in Ghana.

**Methods:**

Mosquito biting densities were established at baseline and during the intervention using human landing collections (HLC). These were carried out overnight from 21:00–06:00 Greenwich Mean Time (GMT). Prior to the HLC, the exposed legs were treated with either a lotion of the 20% IR3535^®^ or 25% DEET (as test) at a rate of 1 g/600 cm^2^, and 70% ethanol (as control). The sampling (HLC) was performed using the Latin square design. Mosquito species were identified morphologically using some keys and molecularly using polymerase chain reaction (PCR). Evaluations were carried out to determine the knock down resistant (*kdr*) allele frequencies. The presence of *Plasmodium falciparum* circumsporozoite proteins was identified using immunological method.

**Results:**

The major malaria vector observed in the study area was *An. gambiae* sensu lato (s.l.) with high frequencies of *kdr-west* mutation in the population. The 20% IR3535^®^ and 25% DEET treatments reduced mosquito bites by 98% and 95%, respectively, compared with the control (*P* < 0.01). The collectors were protected by the 20% IR3535^®^ and 25% DEET for about 92% and 89% of the time, respectively. There was also a sustained protection of the two repellents for 9 h. This protection prevented infectious bites in the treatment group compared with the control.

**Conclusions:**

The 20% IR3535^®^ with Staytec technology can provide significant protection against *Anopheles* and other mosquito bites and will be useful for complementing other vector control interventions.

**Graphical Abstract:**

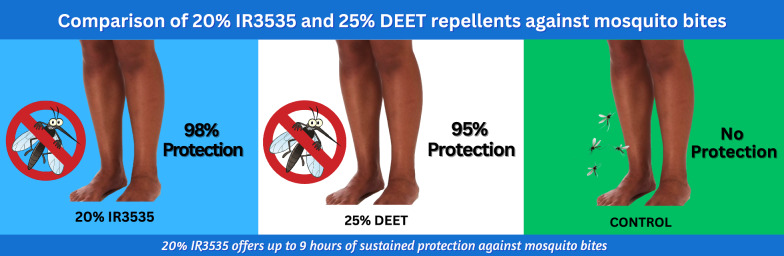

**Supplementary Information:**

The online version contains supplementary material available at 10.1186/s13071-025-06946-1.

## Background

Malaria control in Ghana relies primarily on long-lasting insecticidal nets (LLINs) and indoor residual spraying (IRS) [1]. However, with the recent evolution of pyrethroid resistance and the changing behavior of *Anopheles* mosquitoes (increased outdoor biting) in Ghana [2–6], there are prospects of reducing the efficacy of insecticide-treated nets for preventing disease transmission. These challenges highlight the importance of strengthening the available arsenal of personal protection measures and novel interventions in supporting the malaria elimination agenda in the country.

Topical repellents including DEET have long been known to offer protection against mosquito-borne diseases [7–11]. A recent study indicated that an increase in early evening biting could increase transmission not only because people are unprotected by nets but also because there is a higher chance of malaria vectors becoming infectious [12]. Repellents have been available in developed countries for decades, but their application in less developed countries has been frustrated by doubts about their efficacy, affordability, and user-acceptance [13, 14]. The IR3535^®^ is a technical grade synthetic biochemical pesticide that contains ≥ 99% 3-[*N*-butyl-*N*-acetyl]-aminopropionic acid, ethyl ester as the active ingredient and up to 1.00% inert ingredients. It has been shown to be safe for use on different animals [15–17], including pregnant women and babies [18], and fully biodegradable with no chemical traces left after 48 h. Studies have shown that IR3535^®^ repellents are effective against *Aedes* mosquito species, whereas *Anopheles* mosquitoes seem to be less sensitive to them [11, 13, 14]. The repellent after application lasts between 4 h and 10 h, depending on the formulation, the applied dose, and the mosquito species [13, 14].

The 20% IR3535^®^ with Staytec technology insect repellent formula (LivFul Inc, USA) is a sustained-release formulation of 20% IR3535^®^ (Merck KGaA, Darmstadt, Germany). The proprietary formulation is DEET-free with an emulsion of cosmetic ingredients plus IR3535^®^ that is micro-encapsulated within a polymeric “shell.” It has been designed to reduce the rate of evaporation from the mammalian skin surface and the rate of absorption into the skin. This controlled release results in residual active ingredient remaining on the surface of the skin longer than can be achieved by common emulsions or ointments. Two distinct studies have been performed on this product, which are the laboratory arm-in-cage and field trials. These revealed a minimum limit test (i.e., first confirmed bite) as high as 13–14 h [11]. The repellent is safe for topical use on human skin based upon the various toxicology studies carried out [15–17].

We evaluated the efficacy and protection time of 20% IR3535^®^ lotion (with Staytec technology formulation) in repelling mosquitoes (especially *Anopheles*) in a field setting compared with a 25% DEET lotion. With the growing concern of insecticide resistance in *Anopheles* population and the behavioral change in biting patterns in Ghana [2–6], efficacy studies on repellents are relevant to support personal protection from mosquito bites either indoors or outdoors.

## Methods

The ethical approval for this study was obtained from the Institutional Review Board (IRB) of the Noguchi Memorial Institute for Medical Research (NMIMR) [no. NMIMR-IRB CPN 001/23-24]. The study was carried out in Osrogba and Obom, two villages within the Shai Osudoku District of Ghana. The district capital is Dodowa, a peri-urban area located at 5°53′N, 0°7′E. The inhabitants in Osrogba and Obom are mainly farmers, and their houses are typically made of mud and cement. They have water bodies serving as breeding sites for mosquitoes all year round. Previous work at these sites showed that *An. gambiae* sensu stricto (s.s.) constitutes about 94% of the *Anopheles* population, and *kdr* mutation occurred at high frequencies of about 80–100% [19]. High levels of *GSTe2* gene expression have also been reported in the *An. gambiae* population, with high resistance to dichloro-diphenyl-trichloroethane (DDT) [19].

A baseline biting rate was assessed to establish the densities of mosquitoes in the study areas before testing the repellents. Collections were carried out using standard procedure for human landing catches (HLC) [20] by eight trained mosquito collectors. Each night, hourly collections were performed (with a 10-min break per hour) from four collection points, spaced at least 20 m apart by the eight collectors (one indoor and one outdoor at each point) from 18:00 to 06:00. The collection lasted for four nights from 8 to 11 July 2023 during the baseline.

The trial for the two repellents was carried out from 28 July to 12 August 2023. On each night of the trial, the skin surface area of each collector’s legs was washed with unscented soap and rinsed with water. The area was then rinsed again with a solution of 70% ethanol or isopropyl alcohol and dried with an uncontaminated towel. The length and circumference of the collector’s legs were measured to calculate the surface area (Additional File 1: Supplementary Table S1), and the correct dose of treatment measured. After preparation of the right dosage, the collector’s legs were evenly treated (using a latex glove) between the ankle and the knee with either the 20% IR3535^®^ or 25% DEET lotion at a rate of 1 g/600 cm^2^ (treatment) or with the 70% ethanol (control). The treatment solutions were blinded to the mosquito collectors.

Mosquito collection strategy during the trial followed a Latin square design similar to the baseline collection by the eight trained mosquito collectors (Additional File 2: Supplementary Fig. S1 for the explicit collector rotation scheme). Each night, hourly collections were carried out at the four collection points, which represented the two treatment groups (20% IR3535^®^ and 25% DEET) with their respective controls, all spaced at least 20 m apart. Samples were also segregated by the hour. Collections were carried out in the two villages on alternating days, such that each village was visited every other day. This was done to reduce any residual repellent effect from the previous night’s collection at the collection points. Collections were performed both indoors and outdoors from 21:00 to 06:00 and lasted for 16 nights during the trial. The indoor environments averaged 28 °C and 82% air temperature and humidity, respectively.

All the mosquitoes were transported in a box to the Vector Research laboratory at the NMIMR for species identification, sorting, counting, and laboratory analyses. Adult mosquitoes were identified morphologically using identification keys [21], and the sibling species of *An. gambiae* sensu lato (s.l.) were identified using polymerase chain reaction [22–24]. All the *Anopheles* mosquitoes collected were examined for the presence of *P. falciparum* circumsporozoite protein using a sandwich enzyme-linked immunosorbent assay method as described [25]. This was to assess malaria transmission and the impact of the repellent in reducing the human–vector contact (hence, reduction in sporozoite infectivity of mosquitoes). The *kdr-west* gene mutation (L1014F) in *An. gambiae* s.l. was determined to understand the frequencies in the vector population within the study sites, following the method as described [26].

### Data analysis

Comparison of mosquito landing rates between the control and treated legs were performed using Fisher’s exact test, chi-squared test, and *t*-test analysis using R software (version 4.4.0). Variation in protection time was also measured using the log-rank test analysis. The biting pressure was estimated on the basis of standard World Health Organization (WHO) methods and Kaplan–Meier survival analysis. Percentage reduction in biting rates and sporozoite rates were estimated on the basis of the control collections. Repellency was calculated as %*R* = ([*C* − *T*]/*C*) × 100, where *C* and *T* represent mosquito counts on control and treated legs, respectively. Mean repellency was computed for each test product. The biting rate was estimated as total number of mosquitoes captured/person/night. The entomological inoculation rate (EIR) was calculated as the product of the biting rate (bites/person/night) and the proportion infected with sporozoites.

## Results

At baseline, a total of 616 mosquitoes were collected. *Anopheles gambiae* sensu lato (s.l.) constituted 79.5%, *Culex* species 20.1%, and *Aedes* species 0.4%. The *An. gambiae* s.l. collected biting outdoors were higher than those collected biting indoors (219 indoor versus 271 outdoor). The overall biting rate of mosquitoes during the baseline was 77 bites/person/night. During the intervention, a total of 1118 mosquitoes were collected. *Anopheles gambiae* s.l. constituted 84.5%, *An. funestus* 1.3%, *Culex* species 13.3%, and *Aedes* species 0.9% (Table 1). Out of the *An. gambiae* s.l. identified, *An. coluzzii* constituted about 52.9%, while *An. gambiae* s.s. constituted 47.1%.

**Table 1:** The number of mosquitoes collected stratified by treatment and species during intervention

Mosquito species	Treatment			Total
	20% IR3535^®^	25% DEET	70% ethanol (control)	
*An. gambiae* s.l.	19^a^^***^	53^b^^***^	873^c^	945
*An. funestus*	0^a^^***^	1^b^^***^	13^c^	14
*Culex species*	0^a^^***^	2^b^^***^	147^c^	149
*Aedes species*	0^a^^***^	0^a^^***^	10^b^	10
Total	19	56	1043	1118

^a,b,c^ indicate statistically significant difference between the numbers of each mosquito species collected for the different treatments

^*^indicates the level of statistical significance: ^*^ *P* ≤ 0.05, ^**^ *P* ≤ 0.01, ^***^P ≤ 0.001 tested against the control (70% ethanol) using Fisher’s exact test

Test between 20% IR3535 and 25% DEET for *Aedes* species could not be determined since no mosquito sample was collected

**Fig. 1:**
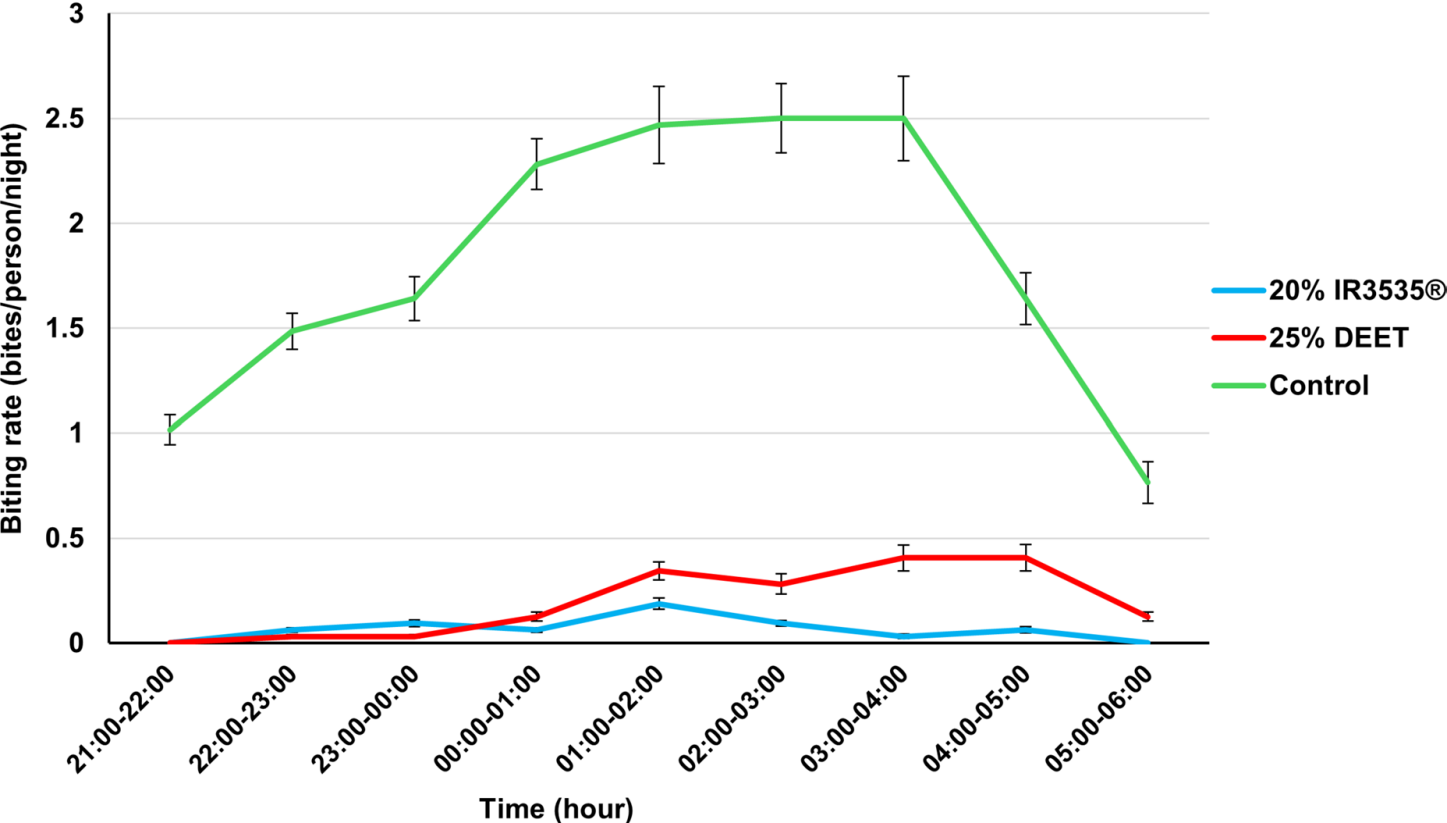
Trends of mosquito biting rates per time for treatments with 20% IR3535^®^, 25% DEET, and 70% ethanol (control). The blue line represents the test treatment 20% IR3535^®^ with Staytec technology, a trade name for an insect repellent called ethyl butylacetylaminopropionate. The red line represents the positive control 25% DEET (*N*,*N*-diethyl-*meta*-toluamide), an active ingredient in some common repellents, and the green line represents the negative control (70% ethanol). The error bars represent the standard deviation of the biting rates (bites/person/night) within the collection days at each time point

The 20% IR3535^®^ treatment reduced mosquito bites by 98% [*t*(8) = 2.31, *P* = 0.003; 95% confidence interval (CI) = 97.5–99.3%], while the 25% DEET treatment reduced bites by 95% [*t*(8) = 2.30, *P* = 0.007; 95% CI = 91.7–98.1%] compared with the control (Table 2). However, there was no significant difference in repellency between the two treatments (*P* = 0.056). The average biting rate during the period for the 20% IR3535^®^ was 0.59 (± 0.83) bites/person/night compared with 1.75 (± 3.43) bites/person/night for 25% DEET and 16.3 (± 11.21) bites/person/night for the control. There was no significant variation between collections indoor and outdoor for the two treatments. The variation in biting rates within the collection days was minimal in the treatment groups than the control, and the peak time of night biting was from 01:00 to 03:00 h (Fig. 1).

**Table 2:** The hourly mosquito landing rate against the 20% IR3535^®^ and 25% DEET

Time (h)	Treatment			% reduction in landing rates for 20% IR3535^®^ treatment	% reduction in landing rates for 25% DEET treatment
	20% IR3535^®^	25% DEET	70% ethanol (control)		
21:00–22:00	0	0	65	100	100
22:00–23:00	2	1	95	98	99
23:00–00:00	3	1	105	97	99
00:00–01:00	2	4	146	99	97
01:00–02:00	6	11	158	96	93
02:00–03:00	3	9	160	98	94
03:00–04:00	1	13	160	99	92
04:00–05:00	2	13	105	98	88
05:00–06:00	0	4	49	100	92
Total	19	56	1043	98	95

The percentage protection time or percentage repellence (% reduction in landing) was calculated on the basis of the formula:

% *R* = ([*C* − *T*]/*C*) × 100

*R* is the repellency or protection time

*C* is the total number of mosquitoes collected on the legs of the control subjects

*T* is the total number of mosquitoes collected on the legs of repellent-treated subjects

Kaplan–Meier survival analysis showed that the average protection time for 20% IR3535^®^ was 91.9% [*P* < 0.001 (*χ*^2^ test); 95% CI = 83.3–95.8%], and for 25% DEET it was 89.2% [*P* < 0.001 (*χ*^2^ test); 95% CI = 86.4–97.8%]. In general, repellent effect waned with time, but the efficacy of both repellents with > 80% lasted for 9 h. The estimates from the risk tables have been shown in the supplementary data (Additional File 3: Supplementary Tables S1–S22).

In determining the sporozoite infectivity, a total of 959 (945 *An. gambiae* s.l. and 14 *An. funestus*) were examined. All mosquitoes from both the 20% IR3535^®^ and 25% DEET treatments were negative (*N* = 73). However, ten samples were positive (1%) from the control treatment collections (*N* = 886). The EIR estimated for the 20% IR3535^®^ and 25% DEET was 0. However, the EIR for the control treatment was 0.163 infective bites/person/night [*t*(884) = 1.96, *P* = 0.003; 95% CI = 0.156–0.164], which translates to about 5 infectious bites monthly and about 60 annually.

In assessing the *kdr-west* frequencies at the Osrogba site, 92% of the genotype was homozygous resistant and 8% was susceptible. The frequency of the mutation was 0.92 (*N* = 25). At the Obom site, 91.3% was homozygous resistant and 8.7% heterozygous resistant, and the frequency of *kdr-west* mutation was 1 (*N* = 23). This was confirmed previously by the high level of pyrethroid and DDT resistance observed in the study areas [6, 27].

## Discussion

Topical repellents have been known to protect people from mosquito bites [28–30]; therefore, new formulations are important for advancing vector control. The 20% IR3535^®^ with Staytec technology insect repellent formula, which is a new formulation of IR3535 with a sustained-release formulation of IR3535^®^ (Merck KGaA, Darmstadt, Germany) and DEET-free, has been evaluated in many countries against several species of mosquitoes [10, 13–15] but not yet in a field setting. We determined the efficacy of this repellent (in comparison with 25% DEET) against mosquitoes, especially *Anopheles* species, in a field setting in southern Ghana**.**

The presence of *An. gambiae* s.l. as the most predominant *Anopheles* species with peak biting time between 01:00–03:00 h in the morning agrees with previous studies that found similar observation in the study area [27]. The two repellents were effective against this species and other mosquito species identified. Although repellent effect of the 20% IR3535^®^ and 25% DEET lotions waned with time, the efficacy of both repellents lasted for over 9 h. Some previous repellent trials conducted in Burkina Faso on a novel repellent estimated a median complete protection time (CPT) of 8 h for 20% DEET and MAÏA repellent [31]. In addition, a community-wide trial on a NO MAS (NM) repellent in northern Ghana estimated an average protective efficacy of 89.6% for 9 h for NM against *Anopheles* mosquitoes [29]. The 20% IR3535^®^ showed an average protective efficacy of 98% for 9 h in the field setting and indicated its superior effectiveness in providing protection to inhabitants in the community. Our study showed a significant reduction in the landing and biting rates of *Anopheles* with the 20% IR3535^®^ and this translated into reduced or no sporozoites detected in the samples examined. Compared with the control, the 20% IR3535^®^ prevented about 60 infectious bites of *Anopheles* mosquitoes within the inhabitants, as inferred from the study’s EIR of 0.163 infective bites/person/night, which translates to about 5 infectious bites monthly and about 60 annually. A previous study in the area estimated annual EIR to be 21 infective bites/person/year [32]. A recent Cochran review of the effect of repellents on malaria reduction indicated that topical repellents may slightly reduce *P. falciparum* prevalence [28].

The high proportion of *An. gambiae* mosquitoes with the *kdr-west* mutation is of great concern, confirming what has already been reported in Ghana [2, 3, 6]. The resistant profile observed emphasizes the very urgent need for other vector control products that can reduce the bites of pyrethroid-resistant mosquitoes. These observations also raise the concerns that pyrethroid-based vector control products such as LLINs for malaria control could be rendered ineffective. Data from this trial indicate that the 20% IR3535^®^ is effective in significantly reducing mosquito bites, including pyrethroid-resistant *Anopheles* mosquitoes. A community-wide trial of the repellent is recommended to determine how this reduction in EIR will translate into malaria reduction.

Comparative studies on the costs between IR3535 and DEET repellents are limited; however, a study has shown the cost of IR3535 to be slightly higher than DEET [33]. This is partly because DEET has been a long-standing standard insect repellent and widely used, while IR3535 is newer and less widely used, accounting for the effect on pricing. Nonetheless, the IR3535 repellents may be preferred for their safety profile [16, 34, 35] and other properties such as being odorless, naturally occurring (biopesticide), less greasy, and cosmetically pleasant for the skin [36]. These characteristics make IR3535 repellents more acceptable among safety-conscious consumers and for cosmetic use.

## Conclusions

This study showed that 20% IR3535^®^ with Staytec technology repellent can provide significant protection against pyrethroid-resistant *Anopheles* and other mosquito bites. This protection can be sustained even in the field setting for about 9 h. The repellent can be an effective addition to complement other vector control interventions such as LLINs and IRS in Ghana.

## Supplementary Information

Additional File 1: Supplementary Table S1. Measurements and amount of repellent applied between the ankle and the knee for each mosquito collector.

Additional File 2: Supplementary Fig. S1. Explicit collector rotation scheme, using a Latin Square Design involving eight trained mosquito collectors using Human Landing Catches.

Additional File 3: Supplementary Tables S1–S22. Survival analysis for DEET and IR3535^®^ with Staytec Technology Repellents (risks summary tables).

## Data Availability

All study data are included in the article.
