# Supplementary material for: Evaluation of the efficacy of 20% IR3535® with a sustained-release formulation and 25% DEET insect repellents against mosquitoes in a field setting in Ghana
Source: Parasit Vectors. 2025 Oct 7;18:398. doi: 10.1186/s13071-025-06946-1 (PMC12505549; doi:10.1186/s13071-025-06946-1)
Supplement: Supplementary file 1 — Additional File 1: Supplementary Table S1. Measurements and amount of repellent applied between the ankle and the knee for each mosquito collector. [file 13071_2025_6946_MOESM1_ESM.docx]

**Additional file 1**

**Table S1.** Measurements and amount of repellent applied between the ankle and the knee for each mosquito collector.

| NAME | Ca(cm) | Ck(cm) | Dak(cm) | Surface Area(cm²) | Mass of Repellent(g) |
| --- | --- | --- | --- | --- | --- |
| Collector A | 36 | 24.5 | 42 | 1270.5 | 2.12 |
| Collector B | 35 | 23.5 | 42 | 1228.5 | 2.05 |
| Collector Cs | 36.5 | 26 | 42 | 1312.5 | 2.19 |
| Collector D | 33.5 | 24.5 | 41 | 1189 | 1.98 |
| Collector E | 37 | 26 | 45 | 1417.5 | 2.36 |
| Collector F | 36.6 | 25 | 40 | 1232 | 2.05 |
| Collector G | 37 | 30 | 42 | 1407 | 2.35 |
| Collector H | 36 | 26 | 43 | 1333 | 2.22 |

Ca = Circumference of the ankle

Ck = Circumference of the knee

Dak = Distance between the ankle and knee.

Measurements were based on WHO protocol: <https://iris.who.int/bitstream/handle/10665/70072/WHO_HTM_NTD_WHOPES_2009.4_eng.pdf;jsessionid=621D64ABF42B103BF739B98F894BE77C?sequence=1>. Accessed 08 May 2025.
