# Supplementary material for: Evaluation of the efficacy of 20% IR3535® with a sustained-release formulation and 25% DEET insect repellents against mosquitoes in a field setting in Ghana
Source: Parasit Vectors. 2025 Oct 7;18:398. doi: 10.1186/s13071-025-06946-1 (PMC12505549; doi:10.1186/s13071-025-06946-1)
Supplement: Supplementary file 2 — Additional File 2: Supplementary Fig. S1. Explicit collector rotation scheme, using a Latin Square Design involving eight trained mosquito collectors using Human Landing Catches. [file 13071_2025_6946_MOESM2_ESM.docx]

**Additional file 2**


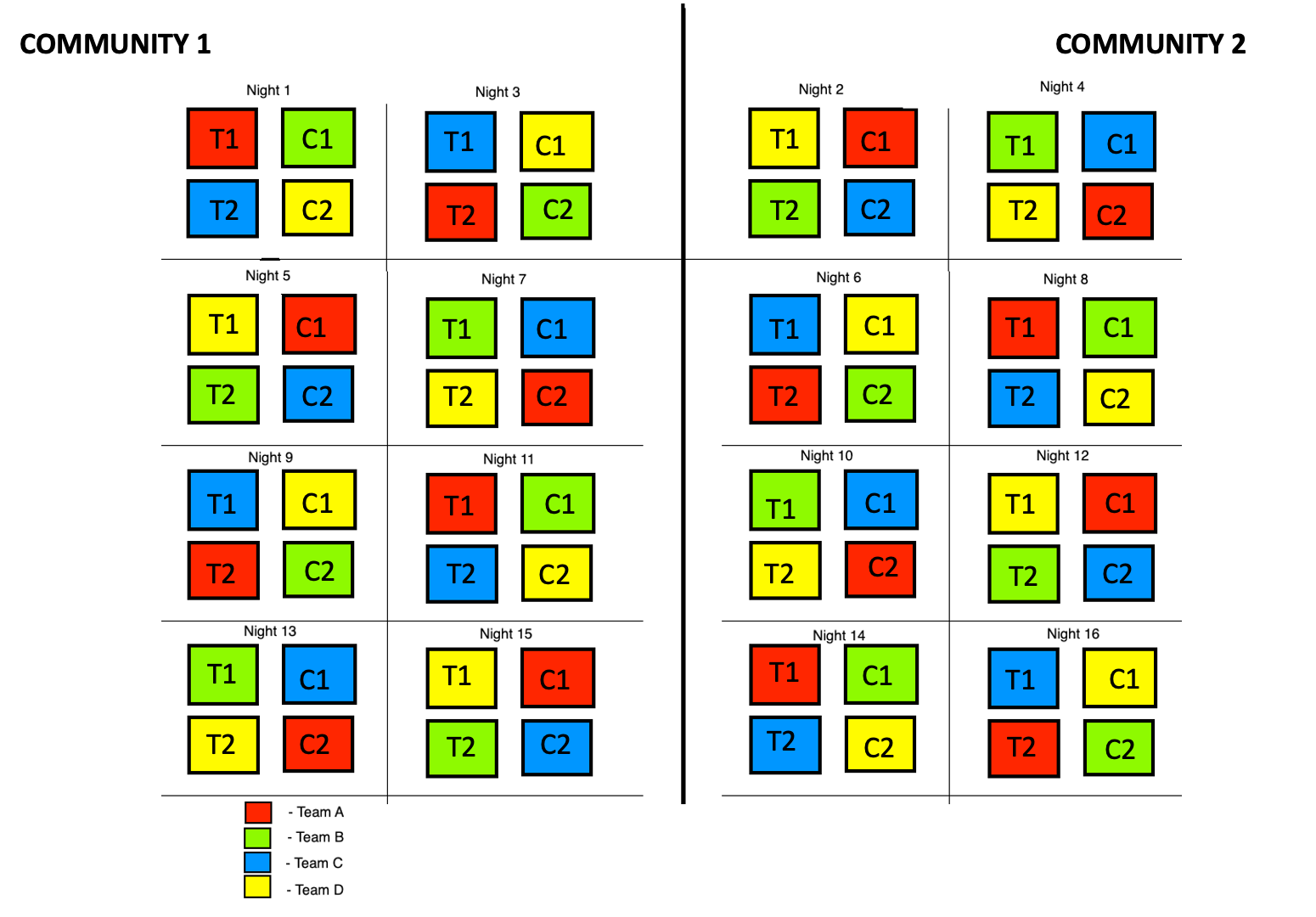


| **No.** | **Team** | **Collector** |
| --- | --- | --- |
| **1** | **A** | Collector 1 |
| **2** |  | Collector 2 |
| **3** | **B** | Collector 3 |
| **4** |  | Collector 4 |
| **5** | **C** | Collector 5 |
| **6** |  | Collector 6 |
| **7** | **D** | Collector 7 |
| **8** |  | Collector 8 |

**Fig. S1.** Explicit collector rotation scheme, using a Latin Square Design for the study. Eight trained mosquito collectors were involved in the collection of mosquitoes using Human Landing Catch (HLC) technique, as shown in the figure above.

Treatment group used either for 20% IR3535^®^ (T1) or 25% DEET (T2) and the Control(C1/C2) group for 70% Ethanol.

| **Test** | **Treatment** |
| --- | --- |
| **T1** | 20% IR3535^®^ |
| **T2** | 25% DEET |
| **C1** | Control 1 (70% Ethanol) |
| **C2** | Control 2 (70% Ethanol) |
