# Supplementary material for: Evaluation of the efficacy of 20% IR3535® with a sustained-release formulation and 25% DEET insect repellents against mosquitoes in a field setting in Ghana
Source: Parasit Vectors. 2025 Oct 7;18:398. doi: 10.1186/s13071-025-06946-1 (PMC12505549; doi:10.1186/s13071-025-06946-1)
Supplement: Supplementary file 3 — Additional File 3: Supplementary Tables S1–S22. Survival analysis for DEET and IR3535® with Staytec Technology Repellents (risks summary tables). [file 13071_2025_6946_MOESM3_ESM.docx]

**Additional file 3: Table S1-S22**

**Survival analysis for DEET and IR3535^®^ with Staytec Technology Repellents (risks summary tables).**

The legends DEET=0, DEET=1, DEET=2, DEET=3, DEET=4, DEET=5, DEET=6, DEET=7, DEET=8 represent the different protection times (hr) for DEET at time 0, 1, 2, 3, 4, 5, 6, 7 and 8 respectively, whereas the times in the respective tables represent the protection times after simulation using the R software. The legends IR3535^®^=1, IR3535^®^=2, IR3535^®^=3 represent the different protection times (hr) for IR3535^®^ at time 1, 2, and 3 respectively, whereas the times in the respective tables represent the protection times after simulation using the R software.

Table S1. Survival analysis for DEET at time 0

| time | n.risk | n.event | survival | std.err | lower 95% CI | Upper 95% CI |
| --- | --- | --- | --- | --- | --- | --- |
| 2 | 108 | 2 | 0.981 | 0.013 | 0.956 | 1 |
| 3 | 93 | 1 | 0.971 | 0.0166 | 0.939 | 1 |
| 4 | 78 | 1 | 0.958 | 0.0205 | 0.919 | 1 |
| 5 | 64 | 2 | 0.929 | 0.0288 | 0.874 | 0.987 |
| 6 | 52 | 2 | 0.893 | 0.0371 | 0.823 | 0.969 |
| 7 | 38 | 1 | 0.869 | 0.043 | 0.789 | 0.958 |
| 8 | 25 | 1 | 0.835 | 0.0535 | 0.736 | 0.946 |
|  |  |  |  |  |  |  |
| Table S2. Survival analysis for DEET at time 1 | | | | | | |
| time | n.risk | n.event | survival | std.err | lower 95% CI | Upper 95% CI |
| 2 | 6 | 1 | 0.833 | 0.152 | 0.583 | 1 |
| 3 | 5 | 1 | 0.667 | 0.192 | 0.379 | 1 |
| 5 | 4 | 1 | 0.5 | 0.204 | 0.225 | 1 |
| 8 | 3 | 3 | 0 | NaN | NA | NA |
|  |  |  |  |  |  |  |
| Table S3. Survival analysis for DEET at time 2 | | | | | | |
| time | n.risk | n.event | survival | std.err | lower 95% CI | Upper 95% CI |
| 4 | 8 | 2 | 0.75 | 0.153 | 0.5027 | 1 |
| 5 | 6 | 1 | 0.625 | 0.171 | 0.3654 | 1 |
| 6 | 5 | 1 | 0.5 | 0.177 | 0.25 | 1 |
| 7 | 4 | 1 | 0.375 | 0.171 | 0.1533 | 0.917 |
| 8 | 3 | 1 | 0.25 | 0.153 | 0.0753 | 0.83 |
| 9 | 2 | 2 | 0 | NaN | NA | NA |
|  |  |  |  |  |  |  |
| Table S4. Survival analysis for DEET at time 3 | | | | | | |
| time | n.risk | n.event | survival | std.err | lower 95% CI | Upper 95% CI |
| 5 | 1 | 1 | 0 | NaN | NA | NA |
|  |  |  |  |  |  |  |
| Table S5. Survival analysis for DEET at time 4 | | | | | | |
| time | n.risk | n.event | survival | std.err | lower 95% CI | Upper 95% CI |
| 5 | 2 | 1 | 0.5 | 0.354 | 0.125 | 1 |
| 7 | 1 | 1 | 0 | NaN | NA | NA |
|  |  |  |  |  |  |  |
| Table S6. Survival analysis for DEET at time 6 | | | | | | |
| time | n.risk | n.event | survival | std.err | lower 95% CI | Upper 95% CI |
| 6 | 2 | 1 | 0.5 | 0.354 | 0.125 | 1 |
| 7 | 1 | 1 | 0 | NaN | NA | NA |
|  |  |  |  |  |  |  |
| Table S7. Survival analysis for DEET at time 8 | | | | | | |
| time | n.risk | n.event | survival | std.err | lower 95% CI | Upper 95% CI |
| 8 | 1 | 1 | 0 | NaN | NA | NA |

Table S8. Survival analysis for IR3535^®^ at time 0

| time | n.risk | n.event | survival | std.err | lower 95% CI | Upper 95% CI |
| --- | --- | --- | --- | --- | --- | --- |
| 2 | 114 | 1 | 0.991 | 0.00873 | 0.974 | 1 |
| 4 | 86 | 2 | 0.968 | 0.01823 | 0.933 | 1 |
| 5 | 71 | 3 | 0.927 | 0.02897 | 0.872 | 0.986 |
| 6 | 58 | 1 | 0.911 | 0.03258 | 0.85 | 0.977 |
| 7 | 45 | 2 | 0.871 | 0.04187 | 0.792 | 0.957 |
| 8 | 31 | 5 | 0.73 | 0.06739 | 0.609 | 0.875 |
| 9 | 16 | 2 | 0.639 | 0.0844 | 0.493 | 0.828 |
|  |  |  |  |  |  |  |
| Table S9. Survival analysis for IR3535^®^ at time 1 | | | | | | |
| time | n.risk | n.event | survival | std.err | lower 95% CI | Upper 95% CI |
| 2 | 10 | 2 | 0.8 | 0.126 | 0.5868 | 1 |
| 3 | 8 | 1 | 0.7 | 0.145 | 0.4665 | 1 |
| 4 | 7 | 1 | 0.6 | 0.155 | 0.3617 | 0.995 |
| 5 | 6 | 1 | 0.5 | 0.158 | 0.269 | 0.929 |
| 6 | 5 | 3 | 0.2 | 0.126 | 0.0579 | 0.691 |
| 7 | 2 | 2 | 0 | NaN | NA | NA |
|  |  |  |  |  |  |  |
| Table S10. Survival analysis for IR3535^®^ at time 2 | | | | | | |
| time | n.risk | n.event | survival | std.err | lower 95% CI | Upper 95% CI |
| 3 | 3 | 1 | 0.667 | 0.272 | 0.2995 | 1 |
| 5 | 2 | 1 | 0.333 | 0.272 | 0.0673 | 1 |
| 8 | 1 | 1 | 0 | NaN | NA | NA |
|  |  |  |  |  |  |  |
| Table S11. Survival analysis for IR3535^®^ at time 3 | | | | | | |
| time | n.risk | n.event | survival | std.err | lower 95% CI | Upper 95% CI |
| 5 | 1 | 1 | 0 | NaN | NA | NA |

Table S12. Survival analysis for DEET at time 0 and IR3535^®^ at time 1

| time | n.risk | n.event | survival | std.err | lower 95% CI | Upper 95% CI |
| --- | --- | --- | --- | --- | --- | --- |
| 2 | 7 | 2 | 0.714 | 0.171 | 0.4471 | 1 |
| 3 | 5 | 1 | 0.571 | 0.187 | 0.3008 | 1 |
| 4 | 4 | 1 | 0.429 | 0.187 | 0.1822 | 1 |
| 6 | 3 | 2 | 0.143 | 0.132 | 0.0233 | 0.877 |
| 7 | 1 | 1 | 0 | NaN | NA | NA |
|  |  |  |  |  |  |  |
| Table S13. Survival analysis for DEET at time 0 and IR3535^®^ at time 2 | | | | | | |
| time | n.risk | n.event | survival | std.err | lower 95% CI | Upper 95% CI |
| 5 | 2 | 1 | 0.5 | 0.354 | 0.125 | 1 |
| 8 | 1 | 1 | 0 | NaN | NA | NA |
|  |  |  |  |  |  |  |
|  |  |  |  |  |  |  |
| Table S14. Survival analysis for DEET at time 0 and IR3535^®^ at time 3 | | | | | | |
| time | n.risk | n.event | survival | std.err | Lower 95% CI | Upper 95% CI |
| 5 | 1 | 1 | 0 | NA | NA | NA |
|  |  |  |  |  |  |  |
| Table S15. Survival analysis for DEET at time 1 and IR3535^®^ at time 0 | | | | | | |
| time | n.risk | n.event | survival | std.err | lower 95% CI | Upper 95% CI |
| 2 | 5 | 1 | 0.8 | 0.179 | 0.516 | 1 |
| 5 | 4 | 1 | 0.6 | 0.219 | 0.293 | 1 |
| 8 | 3 | 3 | 0 | NaN | NA | NA |
|  |  |  |  |  |  |  |
| Table S16. Survival analysis for DEET at time 1 and IR3535^®^ at time 2 | | | | | | |
| time | n.risk | n.event | survival | std.err | lower 95% CI | Upper 95% CI |
| 3 | 1 | 1 | 0 | NaN | NA | NA |
|  |  |  |  |  |  |  |
| Table S17. Survival analysis for DEET at time 2 and IR3535^®^ at time 0 | | | | | | |
| time | n.risk | n.event | survival | std.err | lower 95% CI | Upper 95% CI |
| 4 | 8 | 2 | 0.75 | 0.153 | 0.5027 | 1 |
| 5 | 6 | 1 | 0.625 | 0.171 | 0.3654 | 1 |
| 6 | 5 | 1 | 0.5 | 0.177 | 0.25 | 1 |
| 7 | 4 | 1 | 0.375 | 0.171 | 0.1533 | 0.917 |
| 8 | 3 | 1 | 0.25 | 0.153 | 0.0753 | 0.83 |
| 9 | 2 | 2 | 0 | NaN | NA | NA |
|  |  |  |  |  |  |  |
| Table S18. Survival analysis for DEET at time 3 and IR3535^®^ at time 0 | | | | | | |
| time | n.risk | n.event | survival | std.err | lower 95% CI | Upper 95% CI |
| 5 | 1 | 1 | 0 | NaN | NA | NA |
|  |  |  |  |  |  |  |
| Table S19. Survival analysis for DEET at time 4 and IR3535^®^ at time 0 | | | | | | |
| time | n.risk | n.event | survival | std.err | lower 95% CI | Upper 95% CI |
| 7 | 1 | 1 | 0 | NaN | NA | NA |
|  |  |  |  |  |  |  |
| Table S20. Survival analysis for DEET at time 4 and IR3535^®^ at time 1 | | | | | | |
| time | n.risk | n.event | survival | std.err | lower 95% CI | Upper 95% CI |
| 5 | 1 | 1 | 0 | NaN | NA | NA |
|  |  |  |  |  |  |  |
| Table S21. Survival analysis for DEET at time 6 and IR3535^®^ at time 1 | | | | | | |
| time | n.risk | n.event | survival | std.err | lower 95% CI | Upper 95% CI |
| 6 | 2 | 1 | 0.5 | 0.354 | 0.125 | 1 |
| 7 | 1 | 1 | 0 | NaN | NA | NA |
|  |  |  |  |  |  |  |
| Table S22. Survival analysis for DEET at time 8 and IR3535^®^ at time 0 | | | | | | |
| time | n.risk | n.event | survival | std.err | lower 95% CI | Upper 95% CI |
| 8 | 1 | 1 | 0 | NaN | NA | NA |
